# How COVID-19 lockdown measures could impact patients with bulimia nervosa: Exploratory results from an ongoing experience sampling method study

**DOI:** 10.1192/j.eurpsy.2021.750

**Published:** 2021-08-13

**Authors:** N. Leenaerts, T. Vaessen, J. Ceccarini, E. Vrieze

**Affiliations:** 1 Mind-body Research, Biomedical Sciences Group, KU Leuven, Leuven, Belgium; 2 Center For Contextual Psychiatry, Biomedical Sciences Group, KU Leuven, Leuven, Belgium; 3 Department Of Nuclear Medicine And Molecular Imaging, KU Leuven, Leuven, Belgium

**Keywords:** Experience Sampling Method, COVID-19, Bulimia Nervosa

## Abstract

**Introduction:**

Preliminary results indicate that COVID-19 lockdown measures could lead to an increase in eating disorder pathology. However, some patients could be more vulnerable to experience such an increase than others. The reason why some patients are more susceptible to the impact of lockdown measures is still not known.

**Objectives:**

To analyze the impact of the Belgian COVID-19 lockdown measures on the surroundings, social context, negative affect (NA), positive affect (PA) and binge eating frequency of patients with bulimia nervosa (BN).

**Methods:**

The data of 15 female patients with BN from an ongoing experience sampling method study were analyzed. Mixed effects models compared surroundings, social context, NA, PA and binge eating before and after the implementation of the lockdown measures.

**Results:**

After the implementation of the lockdown measures, significant changes in surroundings and social context were found as well as an increase in NA (p < 0.001) and decrease in PA (p = 0.015). Patients who experienced an increase in binge eating frequency also experienced a stronger increase in NA (p = 0.012) and decrease in PA (p = 0.026) after the lockdown measures were implemented.

**Conclusions:**

Future research should also look at changes in surroundings, social context, affect and how these interact with factors such as personality traits and coping styles when investigating why some patients are more susceptible to the negative effects of lockdown measures than others.

